# Supplementary material for: A comprehensive framework to estimate the frequency, duration, and risk factors for diagnostic delays using bootstrapping-based simulation methods
Source: BMC Med Inform Decis Mak. 2023 Apr 14;23:68. doi: 10.1186/s12911-023-02148-w (PMC10103428; doi:10.1186/s12911-023-02148-w)

**SUPPLEMENTARY MATERIAL**

**Supplementary Methods 1: Assumptions and considerations for estimating baseline trends in the expected number of SSD visits**

In order to apply the bootstrapping techniques summarized in the manuscript, one must first estimate the number of missed opportunities each day during the defined diagnostic opportunity window. Note, only this information is required to implement the bootstrapping techniques. Although there exist a number of ways to estimate and/or define both the number of missed opportunities and bounds on the diagnostic opportunity window, we proposed a number of potential options and considerations for estimating these values based on the hypothesized data generating process outlined in the *Theoretical and Conceptual Framework* subsection of the *Methods* section.

Consideration 1: Regarding the increasing trend prior to the diagnostic opportunity window

In the authors’ previous experience studying diagnostic delays across a range of diseases, (18, 19, 24, 25) we have universally found that the period prior to the diagnostic opportunity window reflects an increasing trend in SSD-related visits. In some cases, such a trend might be driven by the possibility of some missed opportunities occurring outside of the selected diagnostic opportunity window. In such cases, our proposed estimation strategies using an upward trend to estimate the period of visits prior to the diagnostic opportunity window, may tend to underestimate the true number of missed opportunities. This may be particularly true of chronic diseases, those with a long latency period, and in cases where a well-defined biologically plausible window of time defining the diagnostic opportunity window is difficult to identify.

Conversely, we have often observed an increasing trend in SSD-associated visits outside the period of biological plausibility where diagnostic delays can be expected to occur. For example, the figure below depicts the trend in SSD-related visits for fever before a sepsis diagnosis (based on 1,667,360 cases of sepsis that were identified in the MarketScan database that was used for our primary study). As can be seen from this figure, an increasing trend in fever-related visits can be observed for up to a year prior to the index sepsis admission. However, it is not biologically plausible to conclude that patients with sepsis are being misdiagnosed for up to a year before requiring hospitalization for sepsis (given the extremely high fatality rate of sepsis without treatment). Thus, the increase in SSD-related visits over time that occurs outside the diagnostic opportunity window may reflect a variety of factors independent from diagnostic delays. For example, the following factors might explain this increasing trend prior to the diagnostic opportunity window:

1. *Risk factors for the condition of interest* – prior to being diagnosed with a particular disease we might see an increase in healthcare visits for conditions that increase the risk for the disease of interest. Such risk factors might be representing an SSD themself or the visits associated with the risk factor might also coincidentally capture an SSD. For example, diabetes represents a risk factor for TB. We might expect an increase in visits for diabetes-related care before a TB diagnosis, some of which might also coincidentally contain an SSD for TB such as cough or fever.
2. *Deteriorating health* – as patient health declines over time, or as individuals experience periods of poor health, they may be at greater risk for the disease of interest and as a result experience more frequent healthcare visits for SSDs under consideration. For example, patients with a weakened immune system might be at increased risk for TB but may also have an increase in other healthcare visits (attributable to a weakened immune system) prior to developing symptoms of TB.
3. *Surveillance or observation effects* – As patients are observed in healthcare settings for a particular reason X, it may be more likely that unrelated symptoms or diseases Y may be detected simply because of a surveillance effect. Similarly patient healthcare episodes may appear to cluster in time due to factors that increase the likelihood of patient interaction with healthcare settings (e.g., new or changes in insurance enrollment, patient scheduling convenience or when subsequent visits are for follow-up care or in response to disease screening). For example, a patient who recently acquires more generous healthcare coverage may increase the number of healthcare interactions leading to the detection of previously unrecognized symptoms/diseases.
4. *Triggering events* – for some diseases there may exist other triggering events (e.g., diseases, syndromes, stress, other health conditions, etc.) that may trigger the disease of interest. In such cases, care received for the triggering event may appear to increase the number of SSD-visits prior to the index disease diagnosis. For example, a patient may become septic as the result of an initial infection observed in a healthcare encounter. And fever may be considered as an SSD both for the initial infection and the sepsis admission. Thus, fever may appear to increase before sepsis reflecting the triggering event rather than sepsis. Similarly, episodes of physical exertion or stress that may trigger stroke or AMI may result in an increasing pattern of healthcare for the triggering event.

Given the two potential explanations for the increasing trend in SSD-related visits prior to the diagnostic opportunity window (i.e., undetected missed opportunities versus coincidental patterns not directly associated with disease) care must be taken when applying the estimation strategies described in the primary manuscript. Researchers must consider the specific empirical application, whether the bounds of the diagnostic opportunity window can be justified by biologically plausible mechanisms and whether the explanations described above may contribute to an increasing trend prior to the diagnostic opportunity window (e.g., Are their triggering events or other risk factors to consider?). When uncertainty exists, sensitivity analyses of the estimation strategy may need to be considered and the potential for underestimation of the number of missed opportunities may need to be noted.

Increasing trend in fever-related visits over year prior to index sepsis diagnosis

Consideration 2: Regarding the baseline trend during the diagnostic opportunity window

A second aspect to consider is how the unobservable expected baseline trend is estimated during the diagnostic opportunity window. It is important to keep in mind, that the trend during this period represents a counterfactual, which cannot be directly observed. As was depicted in Figure 6, this trend can be estimate using both a linear and non-linear trend. Given a unique change-point, an upward sloping non-linear trend will tend to capture more SSD visits as representing expected care versus a linear trend. Thus, using a non-linear trend when the underlying data-generating process for expected care is linear will tend to result in an underestimate of the number of missed opportunities. Conversely, using a linear-trend when the expected pattern of care should be non-linear will tend to overestimate the number of missed opportunities.

As before, consideration must be given to the empirical application being considered and whether or not there is justification for the trend to be represented as linear or non-linear. When significant uncertainty exists, the investigator may wish to consider a sensitivity analysis using both linear and non-linear models to estimate the trend in expected healthcare utilization. For example, in Supplementary Table 11 we provide a sensitivity analysis of how linear and non-linear trends may impact our resulting estimates for the number of missed opportunities. Such estimates can then be used with the bootstrapping approaches described to provide bounds on the individual delay metrics of interest.

**Supplementary Methods 2: A “prediction bound” approach to find a diagnostic opportunity window change-point using prediction bounds.**

Given the following specification outlined in the main manuscript to describe the trend in SSD visits:

$$y_{t}=\left\{ \begin{aligned} f\left( t \right) t<w \\ g\left( t \right) t\geq w \end{aligned} \right.$$

if one wishes to use the formula $m_{t}=y_{t}-\hat{f}(t)$, described in the main manuscript, to estimate the number of missed opportunities without estimating $g\left( t \right)$, it is possible to find $cp$ by exploiting the fundamental assumption that $y_{t}>f\left( t \right), \forall t>cp.$Specifically, we can define the change-point as the point $cp$ such that $y_{t}> \hat{y}_{t} \forall t>cp$ or using the prediction bound we can define $cp$ such that $y_{t}> \hat{y}_{t}+c\hat{\sigma}_{t} \forall t>cp$, where $c$ is a critical value based on the coverage probability. We can then attempt to find a value of $cp$, by choosing an initial guess $\tilde{cp}\leq cp$ that we believe is outside of the true diagnostic opportunity window, while using the interval $[-T,\tilde{cp]}$ to estimate $f\left( t \right).$ Using this initial guess $\tilde{cp}$, we can then identify the value $cp$ in the problem described above. This change-point-finding approach is universally applicable regardless of the parameterization of $f\left( t \right).$ Of course the performance of this approach is highly dependent on the initial value $\tilde{cp}$, and it is desirable to choose a value as close to the true change point as possible. Note: this approach might be desirable if an upper bound on the feasible diagnostic opportunity window can be clinically justified but where the exact window remains unknown (e.g., diagnostic delays for HSV encephalitis should never exceed 3 months).

**Supplementary Methods 3: Selection of the optimal change-point for the diagnostic opportunity window**

We select the optimal change point based on two criteria intended to balance the assumptions of the data generating process described in the main manuscript. First, the change point approach should maximize the model fit during the period prior to the diagnostic opportunity window, where we intend to estimate the expected trend in SSD visits that will be extrapolated forward into the diagnostic opportunity window. We characterize this trend by choosing the model that minimizes the mean squared error over the period prior to the diagnostic opportunity window. Second, we evaluate the model performance just prior to the change point to ensure that the model does not begin overestimating the trend prior to the delay-opportunity window. Because the observed trend after the change point is expected to monotonically increase, if the change point is set inside the theoretical diagnostic opportunity window we would expect it to result in a negative error as the slope of the expected SSD curve will begin to shift upward. To evaluate this criterion, we evaluate the mean error within 7, 14, and 21 days prior to the change point, and choose the model with a mean error consistently nearest to zero.

**Supplementary Table 1. Disease-specific index diagnosis codes**

| **Disease** | **ICD9-CM** | **ICD10-CM** |
| --- | --- | --- |
| **Stroke** | 430, 431, 432, 432.0, 432.1, 432.9, 433.01, 433.11, 433.21, 433.31, 433.81, 433.91, 434, 434.0, 434.00, 434.01, 434.1, 434.10, 434.11, 434.9, 434.90, 434.91, 346.60, 346.61, 346.62, 346.63, 436, 435, 435.0, 435.1, 435.2, 435.3, 435.8, 435.9 | G43.609, G43.619, G43.601, G43.611, I60.9, I61.9, I62.1, I62.00, I62.9, I63.22, I63.139, I63.239, I63.019, I63.119, I63.219, I63.59, I63.20, I66.09, I66.19, I66.29, I63.30, I66.9, I63.40, I63.50, G45.0, G45.8, G45.1, G45.9, I67.848, I67.89, G45.2, G46.0, G46.1, G46.2, I60.00, I60.01, I60.02, I60.10, I60.11, I60.12, I60.20, I60.21, I60.22, I60.30, I60.31, I60.32, I60.4, I60.50, I60.51, I60.52, I60.6, I60.7, I60.8, I61.0, I61.1, I61.2, I61.3, I61.4, I61.5, I61.6, I61.8, I62.01, I62.02, I62.03, I63.00, I63.011, I63.012, I63.02, I63.031, I63.032, I63.039, I63.09, I63.10, I63.111, I63.112, I63.12, I63.131, I63.132, I63.19, I63.211, I63.212, I63.231, I63.232, I63.29, I63.311, I63.312, I63.319, I63.321, I63.322, I63.329, I63.331, I63.332, I63.339, I63.341, I63.342, I63.349, I63.39, I63.411, I63.412, I63.419, I63.421, I63.422, I63.429, I63.431, I63.432, I63.439, I63.441, I63.442, I63.449, I63.49, I63.511, I63.512, I63.519, I63.521, I63.522, I63.529, I63.531, I63.532, I63.539, I63.541, I63.542, I63.549, I63.6, I63.8, I63.9, I66.01, I66.02, I66.03, I66.11, I66.12, I66.13, I66.21, I66.22, I66.23, I66.3, I66.8, I67.841 |
| **AMI** | 410.0, 410.00, 410.01, 410.02, 410.1, 410.10, 410.11, 410.12, 410.2, 410.20, 410.21, 410.22, 410.3, 410.30, 410.31, 410.32, 410.4, 410.40, 410.41, 410.42, 410.5, 410.50, 410.51, 410.52, 410.6, 410.60, 410.61, 410.62, 410.7, 410.70, 410.71, 410.72, 410.8, 410.80, 410.81, 410.82, 410.9, 410.90, 410.91, 410.92 | I21.01, I21.02, I21.09, I21.11, I21.19, I21.21, I21.29, I21.3, I21.4, I21.9, I21.A1, I21.A9, I22.0, I22.1, I22.2, I22.8, I22.9 |
| **Tuberculosis** | 010, 010.0, 010.00, 010.01, 010.02, 010.03, 010.04, 010.05, 010.06, 010.1, 010.10, 010.11, 010.12, 010.13, 010.14, 010.15, 010.16, 010.8, 010.80, 010.81, 010.82, 010.83, 010.84, 010.85, 010.86, 010.9, 010.90, 010.91, 010.92, 010.93, 010.94, 010.95, 010.96, 011, 011.0, 011.00, 011.01, 011.02, 011.03, 011.04, 011.05, 011.06, 011.1, 011.10, 011.11, 011.12, 011.13, 011.14, 011.15, 011.16, 011.2, 011.20, 011.21, 011.22, 011.23, 011.24, 011.25, 011.26, 011.3, 011.30, 011.31, 011.32, 011.33, 011.34, 011.35, 011.36, 011.4, 011.40, 011.41, 011.42, 011.43, 011.44, 011.45, 011.46, 011.5, 011.50, 011.51, 011.52, 011.53, 011.54, 011.55, 011.56, 011.6, 011.60, 011.61, 011.62, 011.63, 011.64, 011.65, 011.66, 011.7, 011.70, 011.71, 011.72, 011.73, 011.74, 011.75, 011.76, 011.8, 011.80, 011.81, 011.82, 011.83, 011.84, 011.85, 011.86, 011.9, 011.90, 011.91, 011.92, 011.93, 011.94, 011.95, 011.96, 012, 012.0, 012.00, 012.01, 012.02, 012.03, 012.04, 012.05, 012.06, 012.1, 012.10, 012.11, 012.12, 012.13, 012.14, 012.15, 012.16, 012.2, 012.20, 012.21, 012.22, 012.23, 012.24, 012.25, 012.26, 012.3, 012.30, 012.31, 012.32, 012.33, 012.34, 012.35, 012.36, 012.8, 012.80, 012.81, 012.82, 012.83, 012.84, 012.85, 012.86, 018, 018.0, 018.00, 018.01, 018.02, 018.03, 018.04, 018.05, 018.06, 018.8, 018.80, 018.81, 018.82, 018.83, 018.84, 018.85, 018.86, 018.9, 018.90, 018.91, 018.92, 018.93, 018.94, 018.95, 018.96 | A15, A15.0, A15.4, A15.5, A15.6, A15.7, A15.8, A15.9, A19, A19.0, A19.1, A19.2, A19.8, A19.9 |

**Supplementary Table 2. Disease-specific SSD diagnosis codes identified using ICD-9 and ICD-10 diagnosis codes.**

| **Disease** | **SSD** | **ICD-9-CM** | **ICD-10-CM** |
| --- | --- | --- | --- |
| **Stroke** | Headache | 339.00, 339.01, 339.02, 339.03, 339.04, 346.0, 346.00, 346.01, 346.02, 346.03, 346.1, 346.10, 346.11, 346.12, 346.13, 346.2, 346.20, 346.21, 346.22, 346.23, 346.30, 346.31, 346.32, 346.33, 346.40, 346.41, 346.42, 346.43, 346.50, 346.51, 346.52, 346.53, 346.70, 346.71, 346.72, 346.73, 346.8, 346.80, 346.81, 346.82, 346.83, 346.9, 346.90, 346.91, 346.92, 346.93, 339.05, 339.09, 339.10, 339.11, 339.12, 339.20, 339.21, 339.22, 339.3, 339.41, 339.42, 339.43, 339.44, 339.81, 339.82, 339.83, 339.84, 339.85, 339.89, 784.0 | G44.009, G44.019, G44.029, G44.039, G44.049, G44.059, G44.099, G44.209, G44.219, G44.221, G44.229, G44.309, G44.319, G44.329, G44.41, G44.51, G44.52, G44.53, G44.59, G44.81, G44.82, G44.83, G44.84, G44.85, G44.89, G43.109, G43.119, G43.101, G43.111, G43.009, G43.019, G43.001, G43.011, G43.809, G43.A0, G43.B0, G43.C0, G43.D0, G43.819, G43.A1, G43.B1, G43.C1, G43.D1, G43.801, G43.811, G43.409, G43.419, G43.401, G43.411, G43.829, G43.839, G43.821, G43.831, G43.509, G43.519, G43.501, G43.511, G43.709, G43.719, G43.701, G43.711, G43.809, G43.819, G43.801, G43.811, G43.909, G43.919, G43.901, G43.911, G44.1, R51 |
|  | Dizziness | 386.00, 386.01, 386.02, 386.03, 386.04, 386.10, 386.11, 386.12, 386.19, 386.2, 386.30, 386.31, 386.32, 386.33, 386.34, 386.35, 386.40, 386.41, 386.42, 386.43, 386.48, 386.50, 386.51, 386.52, 386.53, 386.54, 386.55, 386.56, 386.58, 386.8, 386.9, 780.4, 380.00, 380.01, 380.02, 380.03, 380.10, 380.11, 380.12, 380.13, 380.14, 380.15, 380.16, 380.21, 380.22, 380.23, 380.30, 380.31, 380.32, 380.39, 380.4, 380.50, 380.51, 380.52, 380.53, 380.81, 380.89, 380.9, 400, 384.01, 384.09, 384.1, 385.30, 385.31, 385.32, 385.33, 385.35, 385.82, 385.83, 385.89, 385.9, 388.00, 388.01, 388.02, 388.10, 388.11, 388.12, 388.2, 388.30, 388.31, 388.32, 388.40, 388.41, 388.42, 388.43, 388.44, 388.45, 388.5, 388.60, 388.61, 388.69, 388.70, 388.71, 388.72, 388.8, 388.9, 389.00, 389.01, 389.02, 389.03, 389.04, 389.05, 389.06, 389.08, 389.10, 389.11, 389.12, 389.13, 389.14, 389.15, 389.16, 389.17, 389.18, 389.2, 389.20, 389.21, 389.22, 389.7, 389.8, 389.9, V41.2, V41.3, V49.85, V53.2, V72.1, V72.11, V72.12, V72.19 | H61.009, H61.019, H61.029, H61.039, H60.00, H60.10, H60.319, H60.329, H60.399, H61.93, H60.339, H62.40, H60.20, H62.8X1, H60.399, H60.40, H60.509, H60.519, H60.529, H60.539, H60.549, H60.559, H60.599, H60.60, H60.8X1, H60.90, H61.109, H61.129, H61.119, H61.199, H61.23, H61.309, H61.399, H61.319, H61.399, H61.329, H61.819, H61.899, H61.93, H73.019, H73.099, H73.10, H71.93, H71.03, H71.13, H74.40, H71.23, H71.33, H74.8X9, H74.8X9, H74.8X9, H74.90, H81.09, H81.09, H81.09, H81.09, H81.09, H81.399, H81.13, H81.23, H81.319, H81.49, H83.09, H83.09, H83.09, H83.09, H83.09, H83.09, H83.19, H83.19, H83.19, H83.19, H83.19, H83.2X9, H83.2X9, H83.2X9, H83.2X9, H83.2X9, H83.2X9, H83.2X9, H83.2X9, H81.8X9, H82.9, H83.8X9, H81.93, H83.93, H93.099, H91.13, H93.019, H83.3X9, H91.8X9, H83.3X9, H91.23, H93.19, H93.19, H93.19, H93.249, H93.299, H93.229, H93.239, H93.299, H93.219, H93.299, H93.3X9, H92.10, G96.0, H92.20, H92.09, H92.09, H92.09, H93.8X9, H93.93, H90.2, H90.2, H90.2, H90.2, H90.2, H90.11, H90.12, H90.0, H90.2, H90.5, H90.3, H90.3, H90.41, H90.42, H90.5, H90.41, H90.42, H90.5, H90.41, H90.42, H90.3, H90.8, H90.71, H90.72, H90.6, H91.3, H91.8X9, H91.90, R42, Z97.4, H93.90, Z73.82, Z46.1, Z01.110, Z01.12, Z01.10, Z01.118 |
| **AMI** | Syncope | 780.2 | R55 |
|  | Other Lower Respiratory Disease | 513.1, 514, 515, 516.0, 516.1, 516.2, 516.3, 516.30, 516.31, 516.32, 516.33, 516.34, 516.35, 516.36, 516.37, 516.4, 516.5, 516.61, 516.62, 516.63, 516.64, 516.69, 516.8, 516.9, 517.2, 517.8, 518.3, 518.4, 518.89, 519.4, 519.8, 519.9, 782.5, 786.00, 786.01, 786.02, 786.03, 786.04, 786.05, 786.06, 786.07, 786.09, 786.2, 786.3, 786.30, 786.31, 786.39, 786.4, 786.52, 786.6, 786.7, 786.8, 786.9, 793.1, 793.11, 793.19, 794.2, V12.6, V12.60, V12.61, V12.69, V42.6 | J182, J22, J810, J811, J82, J8401, J8402, J8403, J8409, J8410, J84111, J84112, J84113, J84114, J84115, J84116, J84117, J8417, J842, J8481, J8482, J8483, J84841, J84842, J84843, J84848, J8489, J849, J852, J853, J984, J986, J988, J989, J99, R042, R0481, R0489, R049, R05, R0600, R0601, R0602, R0603, R0609, R062, R063, R064, R066, R0681, R0682, R0683, R0689, R069, R071, R0781, R0902, R093, R230, R911, R918, Z8701, Z8709, Z942 |
|  | Other Gastrointestinal Disorders | 538, 558.1, 558.2, 564.0, 564.00, 564.01, 564.02, 564.09, 564.1, 564.5, 564.7, 564.8, 564.81, 564.89, 564.9, 568.0, 568.81, 568.82, 568.89, 568.9, 569.81, 569.82, 569.83, 569.84, 569.85, 569.86, 569.87, 569.89, 569.9, 579.0, 579.1, 579.2, 579.8, 579.9, 787.1, 787.2, 787.20, 787.21, 787.22, 787.23, 787.24, 787.29, 787.3, 787.4, 787.5, 787.6, 787.60, 787.61, 787.62, 787.63, 787.7, 787.9, 787.91, 787.99, 789.2, 789.3, 789.30, 789.31, 789.32, 789.33, 789.34, 789.35, 789.36, 789.37, 789.39, 789.4, 789.40, 789.41, 789.42, 789.43, 789.44, 789.45, 789.46, 789.47, 789.49, 789.9, 792.1, 793.4, 793.6, V12.7, V12.70, V12.79, V41.6, V44.1, V44.2, V44.3, V44.4, V45.3, V47.3, V53.5, V53.50, V53.51, V53.59, V55.1, V55.2, V55.3, V55.4 | B5732, K520, K521, K5520, K5521, K5530, K5531, K5532, K5533, K580, K581, K582, K588, K589, K5900, K5901, K5902, K5903, K5904, K5909, K591, K592, K593, K5931, K5939, K598, K599, K631, K632, K633, K634, K6381, K6389, K639, K660, K661, K668, K669, K689, K900, K901, K902, K904, K9049, K9089, K909, K9281, K9289, K929, R1113, R12, R130, R1310, R1311, R1312, R1313, R1314, R1319, R140, R141, R142, R143, R150, R151, R152, R159, R161, R188, R1900, R1901, R1902, R1903, R1904, R1905, R1906, R1907, R1909, R1911, R1912, R1915, R192, R1930, R1931, R1932, R1933, R1934, R1935, R1936, R1937, R194, R195, R197, R198, Z431, Z432, Z433, Z434, Z4651, Z4659, Z8719, Z931, Z932, Z933, Z934, Z9482, Z980, Z9884 |
|  | Nonspecific Chest Pain | 786.50, 786.51, 786.59 | R072, R0782, R0789, R079 |
|  | Malaise and Fatigue | 780.7, 780.71, 780.79 | G933, R530, R531, R5381, R5382, R5383 |
|  | Gastritis and Duodenitis | 535.0, 535.00, 535.01, 535.1, 535.10, 535.11, 535.2, 535.20, 535.21, 535.4, 535.40, 535.41, 535.5, 535.50, 535.51, 535.6, 535.60, 535.61, 535.70, 535.71 | K2900, K2901, K2930, K2931, K2940, K2941, K2950, K2951, K2960, K2961, K2970, K2971, K2980, K2981, K2990, K2991 |
|  | Essential Hypertension | 401.1, 401.9 | I10 |
|  | Esophageal Disorders | 456.1, 456.21, 530.0, 530.1, 530.10, 530.11, 530.12, 530.13, 530.19, 530.2, 530.20, 530.21, 530.3, 530.4, 530.5, 530.6, 530.8, 530.81, 530.83, 530.84, 530.85, 530.89, 530.9 | B5731, I8500, I8510, K200, K208, K209, K210, K219, K220, K2210, K2211, K222, K223, K224, K225, K226, K2270, K22710, K22711, K22719, K228, K229, K23 |
|  | Coronary Atherosclerosis and Other Heart Disease | 411.0, 411.1, 411.8, 411.81, 411.89, 412, 413.0, 413.1, 413.9, 414.0, 414.00, 414.01, 414.06, 414.2, 414.3, 414.4, 414.8, 414.9, V45.81, V45.82 | I200, I201, I208, I209, I237, I240, I241, I248, I249, I2510, I25110, I25111, I25118, I25119, I252, I255, I256, I25750, I25751, I25758, I25759, I25811, I2582, I2583, I2584, I2589, I259, Z951, Z955, Z9861 |
|  | Congestive Heart Failure; Non-Hypertensive | 398.91, 428.0, 428.1, 428.20, 428.21, 428.22, 428.23, 428.30, 428.31, 428.32, 428.33, 428.40, 428.41, 428.42, 428.43, 428.9 | I0981, I501, I5020, I5021, I5022, I5023, I5030, I5031, I5032, I5033, I5040, I5041, I5042, I5043, I50810, I50811, I50812, I50813, I50814, I5082, I5083, I5084, I5089, I509 |
|  | Conditions Associated with Dizziness or Vertigo | 386.00, 386.01, 386.02, 386.03, 386.04, 386.10, 386.11, 386.12, 386.19, 386.2, 386.30, 386.31, 386.32, 386.33, 386.34, 386.35, 386.40, 386.41, 386.42, 386.43, 386.48, 386.50, 386.51, 386.52, 386.53, 386.54, 386.55, 386.56, 386.58, 386.8, 386.9, 780.4 | H8101, H8102, H8103, H8109, H8110, H8111, H8112, H8113, H8120, H8121, H8122, H8123, H81311, H81312, H81313, H81319, H81391, H81392, H81393, H81399, H8141, H8142, H8143, H8149, H818X1, H818X2, H818X3, H818X9, H8190, H8191, H8192, H8193, H821, H822, H823, H829, H8301, H8302, H8303, H8309, H8311, H8312, H8313, H8319, H832X1, H832X2, H832X3, H832X9, H838X1, H838X2, H838X3, H838X9, H8390, H8391, H8392, H8393, R42 |
|  | Cardiac Dysrhythmia | 427.0, 427.1, 427.2, 427.31, 427.32, 427.60, 427.61, 427.69, 427.81, 427.89, 427.9, 785.0, 785.1 | I470, I471, I472, I479, I480, I481, I482, I483, I484, I4891, I4892, I491, I492, I493, I4940, I4949, I495, I498, I499, R000, R001, R002 |
|  | Abdominal Pain | 789.0, 789.00, 789.01, 789.02, 789.03, 789.04, 789.05, 789.06, 789.07, 789.09, 789.60, 789.61, 789.62, 789.63, 789.64, 789.65, 789.66, 789.67, 789.69 | R100, R1010, R1011, R1012, R1013, R102, R1030, R1031, R1032, R1033, R10811, R10812, R10813, R10814, R10815, R10816, R10817, R10819, R10821, R10822, R10823, R10824, R10825, R10826, R10827, R10829, R1084, R109 |
| **Tuberculosis** | Tonsillitis | 463, 474.0, 474.00, 474.01, 474.02, 474.10, 474.11, 475, 474.12, 474.2, 474.8, 474.9 | J03.80, J03.81, J03.90, J03.91, J35.01, J35.02, J35.03, J35.2, J35.3, J35.8, J35.9, J36, J35.1 |
|  | Respiratory Failure | 517.3, 518.81, 518.82, 518.83, 518.84, 799.1 | J96.11, J96.91, R09.01, J80, J96.00, J96.01, J96.02, J96.10, J96.12, J96.20, J96.21, J96.22, J96.90, J96.92 |
|  | Respiratory Cancer | 163.0, 163.1, 163.8, 163.9, 165.0, 165.8, 165.9, 231.1, 231.8, 231.9 | C33, C38.4, C39.0, C39.9, C45.0, D02.1, D02.3, D02.4 |
|  | Pneumonia | 112.4, 114.0, 114.4, 115.05, 115.15, 115.95, 130.4, 136.3, 480.0, 480.1, 480.2, 480.8, 480.9, 481, 482.0, 482.1, 482.2, 482.3, 482.30, 482.31, 482.32, 482.39, 482.4, 482.40, 482.41, 482.42, 482.49, 482.8, 482.81, 482.83, 482.84, 482.89, 482.9, 483, 483.0, 483.1, 483.8, 484.1, 484.3, 484.6, 484.7, 484.8, 485, 486, 513.0, 517.1 | A31.0, A37.01, A37.11, A43.0, A48.1, B25.0, B37.1, B38.0, B38.1, B38.2, B39.0, B39.1, B39.2, B58.3, B59, B77.81, J12.0, J12.1, J12.2, J12.3, J12.89, J12.9, J13, J14, J15.0, J15.1, J15.20, J15.211, J15.212, J15.29, J15.3, J15.4, J15.5, J15.6, J15.7, J15.8, J15.9, J16.0, J16.8, J17, J18.0, J18.1, J18.8, J18.9, J85.1 |
|  | Pleurisy Pneumothorax | 510.0, 511.0, 511.1, 511.8, 511.89, 512.0, 512.8, 512.81, 512.82, 512.83, 512.84, 512.89, 518.1, 518.2, 511.9, 518.0 | J86.0, J93.11, J86.9, J92.0, J92.9, J93.0, J93.12, J93.81, J93.82, J93.83, J94.0, J94.1, J94.2, J94.8, J94.9, J98.19, J98.2, J98.3, R09.1, J90, J91.8, J93.9, J98.11 |
|  | Other Upper-Respiratory Infection | 784.91, 460, 461.0, 461.1, 461.2, 461.3, 461.9, 462, 464.0, 464.00, 464.01, 464.11, 464.20, 464.21, 464.30, 464.31, 464.4, 464.50, 464.51, 465.0, 465.8, 465.9, 473.0, 473.1, 473.2, 473.3, 473.8, 473.9, 464.10 | J00, J01.00, J01.01, J01.10, J01.11, J01.20, J01.21, J01.30, J01.31, J01.40, J01.41, J01.80, J01.81, J01.90, J01.91, J02.0, J02.8, J02.9, J03.00, J03.01, J04.0, J04.10, J04.11, J04.2, J04.30, J04.31, J05.0, J05.10, J05.11, J06.0, J06.9, J32.0, J32.1, J32.2, J32.3, J32.4, J32.8, J32.9, R09.82 |
|  | Other Upper Respiratory Disease | 784.1, 784.41, 784.42, 784.9, 784.99, 472.2, 476.0, 476.1, 478.21, 478.22, 478.24, 478.71, 478.9, 519.2, 472.0, 477.0, 477.2, 477.8, 477.9, 478.1, 478.19, 478.20, 478.29, 478.30, 478.31, 478.32, 478.33, 478.34, 478.4, 478.5, 478.70, 478.74, 478.75, 478.79, 478.8, 519.1, 519.11, 519.19, 519.3, 784.40, 784.49, 784.7, 784.8 | R49.0, R49.8, R49.9, J31.1, J31.2, J39.0, J39.1, J98.09, J98.5, J98.59, R07.0, J30.0, J30.1, J30.2, J30.81, J30.89, J30.9, J31.0, J34.2, J34.89, J37.0, J37.1, J38.00, J38.01, J38.02, J38.1, J38.2, J38.3, J38.4, J38.5, J38.6, J38.7, J39.2, J39.3, J39.8, J39.9, J98.01, J98.51, R04.0, R04.1, R09.81 |
|  | Other Lower Respiratory Disease | 786.02, 786.05, 786.07, 786.2, 786.3, 786.30, 786.4, 786.52, 513.1, 514, 515, 516.0, 516.1, 516.2, 516.3, 516.30, 516.31, 516.32, 516.33, 516.34, 516.35, 516.36, 516.37, 516.4, 516.5, 516.8, 516.9, 517.2, 517.8, 518.3, 518.4, 518.89, 519.4, 519.8, 519.9, 786.00, 786.09, 786.39, 786.9, 793.11, 794.2, 786.6, 786.7, 793.1, 793.19 | R04.2, R05, R06.00, R06.01, R06.02, R06.03, R06.09, R06.2, R06.89, R06.9, R07.1, R07.81, R09.3, J18.2, J22, J85.2, J85.3, J98.9, R06.6, R06.82, J81.0, J81.1, J82, J84.01, J84.02, J84.03, J84.09, J84.10, J84.111, J84.112, J84.113, J84.114, J84.115, J84.116, J84.117, J84.17, J84.2, J84.81, J84.82, J84.89, J84.9, J98.4, J98.6, J98.8, J99, R04.89, R04.9, R09.02, R91.1, R91.8 |
|  | Lung Disease Due to External Agents | 495.0, 495.1, 495.2, 495.3, 495.4, 495.5, 495.6, 495.7, 495.8, 495.9, 500, 501, 502, 503, 504, 505, 506.0, 506.1, 506.2, 506.3, 506.4, 506.9, 507.1, 507.8, 508.0, 508.1, 508.2, 508.8, 508.9 | J60, J61, J62.0, J62.8, J63.0, J63.1, J63.2, J63.3, J63.4, J63.5, J63.6, J64, J66.0, J66.1, J66.2, J66.8, J67.0, J67.1, J67.2, J67.3, J67.4, J67.5, J67.6, J67.7, J67.8, J67.9, J68.0, J68.1, J68.2, J68.3, J68.4, J68.8, J68.9, J69.1, J69.8, J70.0, J70.1, J70.2, J70.3, J70.4, J70.5, J70.8, J70.9 |
|  | Lung Cancer | 162.2, 162.3, 162.4, 162.5, 162.8, 162.9, 209.21 | C34.00, C34.01, C34.02, C34.10, C34.11, C34.12, C34.2, C34.30, C34.31, C34.32, C34.80, C34.81, C34.82, C34.90, C34.91, C34.92, C7A.090, D02.20, D02.21, D02.22 |
|  | Influenza | 487.0, 487.1, 487.8, 488, 488.1, 488.11, 488.12, 488.19, 488.81, 488.82, 488.89 | J09.X1, J09.X2, J09.X3, J09.X9, J10.00, J10.01, J10.08, J10.1, J10.2, J10.89, J11.00, J11.08, J11.1, J11.2, J11.81, J11.82, J11.83, J11.89 |
|  | Hemoptysis | 786.3, 786.30, 786.39 | R04.2, R04.8, R04.89, R04.9 |
|  | Fever | 780.6, 780.60, 780.61 | R50, R50.81, R50.9 |
|  | Cough | 786.2 | R05 |
|  | COPD | 490, 491.0, 491.1, 491.8, 491.9, 491.2, 491.20, 491.21, 491.22, 492.0, 492.8, 494, 494.0, 494.1, 496 | J40, J41.0, J41.1, J42, J44.0, J47.0, J41.8, J43.0, J43.1, J43.2, J43.8, J43.9, J44.1, J44.9, J47.1, J47.9 |
|  | Bronchitis | 466.0, 466.1, 466.11, 466.19 | J20.0, J20.1, J20.2, J20.3, J20.4, J20.5, J20.6, J20.7, J20.8, J20.9, J21.0, J21.1, J21.8, J21.9 |
|  | Asthma | 493.00, 493.01, 493.02, 493.10, 493.11, 493.12, 493.20, 493.21, 493.22, 493.81, 493.82, 493.90, 493.92 | J45.20, J45.21, J45.22, J45.30, J45.31, J45.32, J45.40, J45.41, J45.42, J45.50, J45.51, J45.52, J45.901, J45.902, J45.909, J45.990, J45.991, J45.998 |
|  | Aspiration Pneumonitis | 507 | J69.0 |
|  | Additional Codes | 780.79, 780.8, 783.21, 786.50, 786.51, 786.59, 038.9, 079.99, 310, 340, 391, 599.0, 830, 995.91, 135, 197.0, 212.3, 235.7, 239.1, 289.1, 416.8, 423.9, 428.0, 446.4, 263.9, 276.1, 285.29, 285.9, 288.60, 289.3, 429.3, 782.2, 784.2, 785.0, 785.6, 799.02, 799.4 | R07.2, R07.82, R07.89, R07.9, R53.1, R53.81, R53.83, R61, R63.4, A41.9, B34.9, D14.30, D38.1, D49.1, D86.0, D86.9, I50.9, J85.0, N39.0, D64.9, D72.829, E871, I51.7, R00.0, R22.0, R22.1, R22.2, R59, R59.0, R59.1, R59.9 |

**Supplementary Table 3. Change-point, delay window, and number of missed opportunities by change-point detection approach and disease**

|  | **Stroke** | | | **AMI** | | | **Tuberculosis** | | |
| --- | --- | --- | --- | --- | --- | --- | --- | --- | --- |
|  | ***Linear- Cubic*** | ***CUSUM*** | ***Prediction Bound*** | ***Linear- Cubic*** | ***CUSUM*** | ***Prediction Bound*** | ***Linear- Cubic*** | ***CUSUM*** | ***Prediction Bound*** |
| **Change-point** | 8 | 30 | 39 | 4 | 36 | 40 | 114 | 78 | 74 |
| **Delay Window** | [8, 1] | [30, 1] | [39, 1] | [4, 1] | [36, 1] | [40, 1] | [114, 1] | [78, 1] | [74, 1] |
| **Total Number of Missed Opportunities During Delay Window (% of SSD visits during delay window)** | 28,630 (77.4%) | 40,034 (64.8%) | 41,577 (60.5%) | 65,025 (84.35%) | 116,209 (48.71%) | 117,344 (46.24%) | 6,444 (58.1%) | 6,247 (65.2%) | 6,068 (65.0%) |

**Supplementary Table 4. Assessment of fit by change-point detection approach** – we identified the change-point approach that appeared to best fit the pattern of SSD visits based on the following criteria: (1) minimal MSE before the change-point, (2) mean error right before the change-point that is near zero.

| **Disease** | **Method** | **MSE Before**  **Change-point** | **Mean error X days before change-point** | | | |
| --- | --- | --- | --- | --- | --- | --- |
|  |  |  | ***7-days*** | ***14-Days*** | ***21-Days*** | ***28-Days*** |
| **Stroke** | Linear Cubic | 23319.6 | 533.7 | 310.3 | 197.4 | 121.5 |
|  | CUSUM | 1759.1 | 102.8 | 72.5 | 55.1 | 41.8 |
|  | Prediction Bound | 1311.0 | 60.0 | 42.0 | 28.1 | 16.8 |
| **AMI** | Linear Cubic | 436860.9 | 2512.3 | 1603.1 | 1067.9 | 725.9 |
|  | CUSUM | 19459.8 | 213.9 | 171.8 | 133.0 | 99.8 |
|  | Prediction Bound | 19484.8 | 180.0 | 125.2 | 109.4 | 66.6 |
| **Tuberculosis** | Linear Cubic | 44.4 | -2.6 | -0.9 | -0.5 | 0.3 |
|  | CUSUM | 54.1 | 6.8 | 6.7 | 7.5 | 5.3 |
|  | Prediction Bound | 61.1 | 9.9 | 7.9 | 7.5 | 6.2 |

Note: ME = Mean Error; MSE = Mean Squared Error

**Supplementary Table 5. Estimates of the number of missed opportunities each day during the diagnostic opportunity window used in the bootstrapping models.** The % of all visits on a given day that were estimated to be a missed opportunity are also given.

| **Days Prior to Index** | **Stroke** | | **AMI** | | **Tuberculosis** | |
| --- | --- | --- | --- | --- | --- | --- |
|  | Number of Missed Opportunities | % of all visits | Number of Missed Opportunities | % of all visits | Number of Missed Opportunities | % of all visits |
| 1 | 12472 | 94.5 | 45950 | 91.2 | 272 | 85.5 |
| 2 | 5350 | 88.1 | 10981 | 71.1 | 247 | 84.3 |
| 3 | 3561 | 83.2 | 7147 | 61.6 | 189 | 80.4 |
| 4 | 2788 | 79.5 | 5674 | 56.1 | 192 | 80.7 |
| 5 | 2322 | 76.4 | 4817 | 52.1 | 155 | 77.1 |
| 6 | 1817 | 71.7 | 4416 | 49.9 | 215 | 82.4 |
| 7 | 1592 | 69.0 | 4258 | 49.1 | 253 | 84.6 |
| 8 | 1320 | 64.9 | 3199 | 42.0 | 191 | 80.6 |
| 9 | 1028 | 59.1 | 2508 | 36.3 | 153 | 76.9 |
| 10 | 837 | 54.1 | 2287 | 34.2 | 137 | 75.3 |
| 11 | 785 | 52.5 | 2013 | 31.4 | 138 | 75.4 |
| 12 | 680 | 49.0 | 1974 | 31.0 | 118 | 72.4 |
| 13 | 692 | 49.5 | 2123 | 32.6 | 164 | 78.5 |
| 14 | 715 | 50.4 | 2098 | 32.4 | 203 | 81.9 |
| 15 | 654 | 48.2 | 1637 | 27.2 | 133 | 74.7 |
| 16 | 415 | 37.2 | 1227 | 21.9 | 106 | 70.2 |
| 17 | 372 | 34.7 | 1228 | 22.0 | 125 | 73.5 |
| 18 | 308 | 30.6 | 1091 | 20.0 | 100 | 69.0 |
| 19 | 311 | 30.9 | 1020 | 19.0 | 78 | 63.4 |
| 20 | 298 | 30 | 1122 | 20.5 | 128 | 74.0 |
| 21 | 359 | 34.1 | 1418 | 24.6 | 162 | 78.3 |
| 22 | 317 | 31.4 | 918 | 17.5 | 142 | 76.3 |
| 23 | 279 | 28.8 | 634 | 12.8 | 94 | 68.1 |
| 24 | 188 | 21.4 | 586 | 11.9 | 77 | 63.6 |
| 25 | 154 | 18.3 | 707 | 14.1 | 65 | 59.6 |
| 26 | 189 | 21.6 | 728 | 14.5 | 100 | 69.4 |
| 27 | 238 | 25.8 | 778 | 15.3 | 90 | 67.2 |
| 28 | 322 | 32.1 | 934 | 17.9 | 123 | 73.7 |
| 29 | 181 | 21 | 644 | 13.1 | 102 | 69.9 |
| 30 | 214 | 24 | 379 | 8.1 | 80 | 64.5 |
| 31 | 135 | 16.6 | 340 | 7.4 | 75 | 63.0 |
| 32 | 42 | 5.8 | 238 | 5.3 | 72 | 62.1 |
| 33 | 114 | 14.4 | 281 | 6.2 | 71 | 62.3 |
| 34 | 142 | 17.4 | 470 | 9.9 | 80 | 65 |
| 35 | 143 | 17.5 | 669 | 13.6 | 96 | 69.1 |
| 36 | 125 | 15.7 | 326 | 7.1 | 85 | 66.4 |
| 37 | 70 | 9.5 | 172 | 3.9 | 63 | 59.4 |
| 38 | 15 | 2.2 | 160 | 3.6 | 52 | 54.7 |
| 39 | 33 | 4.7 | 33 | 0.8 | 43 | 50.0 |
| 40 | - | - | 159 | 3.6 | 73 | 62.9 |
| 41 | - | - | - | - | 78 | 64.5 |
| 42 | - | - | - | - | 88 | 67.2 |
| 43 | - | - | - | - | 41 | 48.8 |
| 44 | - | - | - | - | 49 | 53.3 |
| 45 | - | - | - | - | 29 | 40.8 |
| 46 | - | - | - | - | 34 | 44.7 |
| 47 | - | - | - | - | 25 | 37.3 |
| 48 | - | - | - | - | 62 | 59.6 |
| 49 | - | - | - | - | 70 | 62.5 |
| 50 | - | - | - | - | 57 | 57.6 |
| 51 | - | - | - | - | 26 | 38.2 |
| 52 | - | - | - | - | 15 | 26.3 |
| 53 | - | - | - | - | 38 | 47.5 |
| 54 | - | - | - | - | 37 | 46.8 |
| 55 | - | - | - | - | 33 | 44.0 |
| 56 | - | - | - | - | 63 | 60.0 |
| 57 | - | - | - | - | 39 | 48.8 |
| 58 | - | - | - | - | 22 | 34.9 |
| 59 | - | - | - | - | 31 | 43.1 |
| 60 | - | - | - | - | 8 | 16.3 |
| 61 | - | - | - | - | 11 | 21.2 |
| 62 | - | - | - | - | 26 | 38.8 |
| 63 | - | - | - | - | 44 | 51.8 |
| 64 | - | - | - | - | 27 | 39.7 |
| 65 | - | - | - | - | 16 | 28.1 |
| 66 | - | - | - | - | 22 | 34.9 |
| 67 | - | - | - | - | 10 | 19.6 |
| 68 | - | - | - | - | 15 | 27.3 |
| 69 | - | - | - | - | 15 | 27.3 |
| 70 | - | - | - | - | 30 | 42.9 |
| 71 | - | - | - | - | 30 | 42.9 |
| 72 | - | - | - | - | 7 | 14.9 |
| 73 | - | - | - | - | 1 | 2.4 |
| 74 | - | - | - | - | 10 | 20.0 |
| 75 | - | - | - | - | 0 | 0 |
| 76 | - | - | - | - | 31 | 43.7 |
| 77 | - | - | - | - | 27 | 40.3 |
| 78 | - | - | - | - | 24 | 37.5 |
| 79 | - | - | - | - | 0 | 0 |
| 80 | - | - | - | - | 2 | 4.9 |
| 81 | - | - | - | - | 0 | 0 |
| 82 | - | - | - | - | 0 | 0 |
| 83 | - | - | - | - | 9 | 18.8 |
| 84 | - | - | - | - | 31 | 44.3 |
| 85 | - | - | - | - | 4 | 9.3 |
| 86 | - | - | - | - | 0 | 0 |
| 87 | - | - | - | - | 0 | 0 |
| 88 | - | - | - | - | 21 | 35.0 |
| 89 | - | - | - | - | 1 | 2.5 |
| 90 | - | - | - | - | 23 | 37.1 |
| 91 | - | - | - | - | 6 | 13.3 |
| 92 | - | - | - | - | 3 | 7.3 |
| 93 | - | - | - | - | 15 | 28.3 |
| 94 | - | - | - | - | 6 | 13.6 |
| 95 | - | - | - | - | 0 | 0 |
| 96 | - | - | - | - | 11 | 22.4 |
| 97 | - | - | - | - | 5 | 11.6 |
| 98 | - | - | - | - | 24 | 38.7 |
| 99 | - | - | - | - | 4 | 9.5 |
| 100 | - | - | - | - | 0 | 0 |
| 101 | - | - | - | - | 3 | 7.3 |
| 102 | - | - | - | - | 0 | 0 |
| 103 | - | - | - | - | 7 | 15.9 |
| 104 | - | - | - | - | 0 | 0 |
| 105 | - | - | - | - | 6 | 14 |
| 106 | - | - | - | - | 0 | 0 |
| 107 | - | - | - | - | 0 | 0 |
| 108 | - | - | - | - | 3 | 7.5 |
| 109 | - | - | - | - | 0 | 0 |
| 110 | - | - | - | - | 0 | 0 |
| 111 | - | - | - | - | 6 | 14.0 |
| 112 | - | - | - | - | 15 | 28.8 |
| 113 | - | - | - | - | 6 | 14.0 |
| 114 | - | - | - | - | 0 | 0 |

**Supplementary Table 6. Expanded Simulation results for stroke**

|  | **Algorithm 1** | **Algorithm 2 (alpha = 0)** | **Algorithm 2 (alpha = 1)** |
| --- | --- | --- | --- |
| **Number of Missed Opportunities (n 95% CI))** |  |  |  |
| Outpatient | 26042 (62.6%) [CI: 25951 -26144 (62.4 - 62.9%)] | 26211 (63.0%) [CI: 26109 - 26317 (62.8 - 63.3%)] | 25579 (61.5%) [CI: 25502 - 25656 (61.3 - 61.7%)] |
| Inpatient | 1815 (4.4%) [CI: 1765 - 1861 (4.2 - 4.5%)] | 1771 (4.3%) [CI: 1725 - 1818 (4.1 - 4.4%)] | 1871 (4.5%) [CI: 1831 - 1915 (4.4 - 4.6%)] |
| ED | 13720 (33.0%) [CI: 13631 -13807 (32.8 - 33.2%)] | 13595 (32.7%) [CI: 13500 - 13688 (32.5 - 32.9%)] | 14127 (34%) [CI: 14054 - 14203 (33.8 - 34.2%)] |
| Total | 41577 | 41577 | 41577 |
| **Number of Patients Experiencing Missed Opportunities (n (% of all patients) [95% CI])** |  |  |  |
| 0 | 340347 (92.5%) [340258 - 340437 (92.5 - 92.6%)] | 342268 (93.1%) [342169 - 342368 (93.0 - 93.1%)] | 337272 (91.7%) [337215 - 337326 (91.7 - 91.7%)] |
| ≥ 1 | 27421 (7.5%) [27331 - 27510 (7.4 - 7.5%)] | 25500 (6.9%) [25400 - 25599 (6.9 - 7.0%)] | 30496 (8.3%) [30442 - 30553 (8.3 - 8.3%)] |
| ≥ 2 | 8748 (2.4%) [8679 - 8819 (2.4 - 2.4%)] | 8680 (2.4%) [8606 - 8751 (2.3 - 2.4%)] | 8148 (2.2%) [8091 - 8204 (2.2 - 2.2%)] |
| ≥ 3 | 3122 (0.8%) [3074 - 3175 (0.8 - 0.9%)] | 3526 (1.0%) [3474 - 3580 (0.9 - 1.0%)] | 2138 (0.6%) [2103 - 2174 (0.6 - 0.6%)] |
| ≥ 4 | 1239 (0.3%) [1201 - 1277 (0.3 - 0.3%)] | 1653 (0.4%) [1613 - 1696 (0.4 - 0.5%)] | 572 (0.2%) [545 - 599 (0.1 - 0.2%)] |
| ≥ 5 | 539 (0.1%) [512 - 567 (0.1 - 0.2%)] | 861 (0.2%) [830 - 893 (0.2 - 0.2%)] | 163 (0.0%) [147 - 180 (0.0 - 0.0%)] |
| Mean - Overall | 1.52 [1.51 - 1.52] | 1.63 [1.62 - 1.64] | 1.36 [1.36 - 1.37] |
| Median - Overall | 1 [1 - 1] | 1 [1 - 1] | 1 [1 - 1] |
| Mean - Outpatient | 0.95 [0.95 - 0.95] | 1.03 [1.02 - 1.03] | 0.84 [0.84 - 0.84] |
| Median - Outpatient | 1 [1 - 1] | 1 [1 - 1] | 1 [1 - 1] |
| Mean - Inpatient | 0.07 [0.06 - 0.07] | 0.07 [0.07 - 0.07] | 0.06 [0.06 - 0.06] |
| Median - Inpatient | 0 [0 - 0] | 0 [0 - 0] | 0 [0 - 0] |
| Mean - ED | 0.50 [0.50 - 0.50] | 0.53 [0.53 - 0.54] | 0.46 [0.46 - 0.47] |
| Median - ED | 0 [0 - 0] | 0 [0 - 0] | 0 [0 - 0] |
| **Durations of Delays (n (% of patients experiencing missed opportunities) [95% CI])** |  |  |  |
| ≥ 1 days | 27421 (100.0%) [27331 - 27510 (100.0 - 100.0%)] | 25500 (100.0%) [25400 - 25599 (100.0 - 100.0%)] | 30496 (100.0%) [30442 - 30553 (100.0 - 100.0%)] |
| ≥ 3 days | 16047 (58.5%) [15966 - 16126 (58.3 - 58.7%)] | 14107 (55.3%) [14006 - 14196 (55.1 - 55.5%)] | 18841 (61.8%) [18792 - 18891 (61.7 - 61.9%)] |
| ≥ 7 days | 9899 (36.1%) [9827 - 9967 (35.9 - 36.3%)] | 8189 (32.1%) [8106 - 8266 (31.8 - 32.4%)] | 11879 (39.0%) [11835 - 11920 (38.8 - 39.1%)] |
| ≥ 11 days | 6775 (24.7%) [6717 - 6833 (24.5 - 24.9%)] | 5405 (21.2%) [5328 - 5474 (20.9 - 21.4%)] | 7882 (25.8%) [7846 - 7919 (25.8 - 25.9%)] |
| ≥ 15 days | 4714 (17.2%) [4667 - 4760 (17.0 - 17.4%)] | 3680 (14.4%) [3615 - 3739 (14.2 - 14.6%)] | 5316 (17.4%) [5287 - 5345 (17.3 - 17.5%)] |
| ≥ 19 days | 3366 (12.3%) [3330 - 3402 (12.1 - 12.4%)] | 2614 (10.2%) [2559 - 2666 (10.0 - 10.4%)] | 3709 (12.2%) [3687 - 3730 (12.1 - 12.2%)] |
| ≥ 22 days | 2580 (9.4%) [2549 - 2609 (9.3 - 9.5%)] | 1834 (7.2%) [1789 - 1877 (7.0 - 7.4%)] | 2493 (8.2%) [2476 - 2510 (8.1 - 8.2%)] |
| ≥ 26 days | 1793 (6.5%) [1770 - 1816 (6.5 - 6.6%)] | 1361 (5.3%) [1326 - 1395 (5.2 - 5.5%)] | 1717 (5.6%) [1704 - 1730 (5.6 - 5.7%)] |
| Mean - Among Delayed | 7.41 [7.38 - 7.44] | 6.72 [6.67 - 6.76] | 7.64 [7.63 - 7.66] |
| Median - Among Delayed | 4 [4 - 4] | 3 [3 - 3] | 4 [4 - 4] |
| Mean - Everyone Included | 0.55 [0.55 - 0.56] | 0.47 [0.46 - 0.47] | 0.63 [0.63 - 0.64] |
| Median - Everyone Included | 0 [0 - 0] | 0 [0 - 0] | 0 [0 - 0] |

**Supplementary Table 7. Expanded simulation results for AMI**

|  | **Algorithm 1** | **Algorithm 2 (alpha = 0)** | **Algorithm 2 (alpha = 1)** |
| --- | --- | --- | --- |
| **Number of Missed Opportunities (n 95% CI))** |  |  |  |
| Outpatient | 89172 (76%) [CI: 88992-89340 (75.8-76.1%)] | 89826 (76.5%) [CI: 89642-90028 (76.4-76.7%)] | 87726 (74.8%) [CI: 87581-87879 (74.6-74.9%)] |
| Inpatient | 6655 (5.7%) [CI: 6549-6754 (5.6-5.8%)] | 6624 (5.6%) [CI: 6529-6729 (5.6-5.7%)] | 6662 (5.7%) [CI: 6569-6757 (5.6-5.8%)] |
| ED | 21518 (18.3%) [CI: 21374-21666 (18.2-18.5%)] | 20895 (17.8%) [CI: 20734-21057 (17.7-17.9%)] | 22956 (19.6%) [CI: 22826-23085 (19.5-19.7%)] |
| Total | 117344 | 117344 | 117344 |
| **Number of Patients Experiencing Missed Opportunities (n (% of all patients) [95% CI])** |  |  |  |
| 0 | 284618 (79.1%) [284432 - 284799 (79.1 - 79.2%)] | 294841 (82.0%) [294642 - 295041 (81.9 - 82.0%)] | 273044 (75.9%) [272928 - 273162 (75.9 - 76.0%)] |
| ≥ 1 | 75007 (20.9%) [74826 - 75193 (20.8 - 20.9%)] | 64784 (18.0%) [64584 - 64983 (18.0 - 18.1%)] | 86581 (24.1%) [86463 - 86697 (24.0 - 24.1%)] |
| ≥ 2 | 27859 (7.7%) [27723 - 27990 (7.7 - 7.8%)] | 26912 (7.5%) [26774 - 27062 (7.4 - 7.5%)] | 24065 (6.7%) [23955 - 24167 (6.7 - 6.7%)] |
| ≥ 3 | 9268 (2.6%) [9160 - 9374 (2.5 - 2.6%)] | 11279 (3.1%) [11165 - 11386 (3.1 - 3.2%)] | 5435 (1.5%) [5371 - 5505 (1.5 - 1.5%)] |
| ≥ 4 | 3158 (0.9%) [3084 - 3233 (0.9 - 0.9%)] | 5475 (1.5%) [5389 - 5556 (1.5 - 1.5%)] | 1024 (0.3%) [986 - 1062 (0.3 - 0.3%)] |
| ≥ 5 | 1170 (0.3%) [1120 - 1220 (0.3 - 0.3%)] | 3024 (0.8%) [2960 - 3090 (0.8 - 0.9%)] | 195 (0.1%) [175 - 214 (0.0 - 0.1%)] |
| Mean - Overall | 1.56 [1.56 - 1.57] | 1.81 [1.81 - 1.82] | 1.36 [1.35 - 1.36] |
| Median - Overall | 1 [1 - 1] | 1 [1 - 1] | 1 [1 - 1] |
| Mean - Outpatient | 1.19 [1.19 - 1.19] | 1.39 [1.38 - 1.39] | 1.01 [1.01 - 1.02] |
| Median - Outpatient | 1 [1 - 1] | 1 [1 - 1] | 1 [1 - 1] |
| Mean - Inpatient | 0.09 [0.09 - 0.09] | 0.10 [0.10 - 0.10] | 0.08 [0.08 - 0.08] |
| Median - Inpatient | 0 [0 - 0] | 0 [0 - 0] | 0 [0 - 0] |
| Mean - ED | 0.29 [0.28 - 0.29] | 0.32 [0.32 - 0.33] | 0.27 [0.26 - 0.27] |
| Median - ED | 0 [0 - 0] | 0 [0 - 0] | 0 [0 - 0] |
| **Durations of Delays (n (% of patients experiencing missed opportunities) [95% CI])** |  |  |  |
| ≥ 1 days | 75007 (100.0%) [74826 - 75193 (100.0 - 100.0%)] | 64784 (100.0%) [64584 - 64983 (100.0 - 100.0%)] | 86581 (100.0%) [86463 - 86697 (100.0 - 100.0%)] |
| ≥ 3 days | 42701 (56.9%) [42551 - 42863 (56.8 - 57.1%)] | 27716 (42.8%) [27536 - 27899 (42.6 - 43.0%)] | 47940 (55.4%) [47853 - 48023 (55.3 - 55.4%)] |
| ≥ 7 days | 29622 (39.5%) [29495 - 29753 (39.4 - 39.6%)] | 17959 (27.7%) [17794 - 18130 (27.5 - 27.9%)] | 32084 (37.1%) [32012 - 32150 (37.0 - 37.1%)] |
| ≥ 11 days | 21411 (28.5%) [21305 - 21519 (28.4 - 28.7%)] | 13033 (20.1%) [12892 - 13177 (19.9 - 20.3%)] | 23047 (26.6%) [22991 - 23102 (26.6 - 26.7%)] |
| ≥ 15 days | 15391 (20.5%) [15312 - 15469 (20.4 - 20.6%)] | 9012 (13.9%) [8884 - 9137 (13.7 - 14.1%)] | 15761 (18.2%) [15721 - 15801 (18.2 - 18.3%)] |
| ≥ 19 days | 11285 (15.0%) [11217 - 11344 (15.0 - 15.1%)] | 6895 (10.6%) [6790 - 7010 (10.5 - 10.8%)] | 11386 (13.2%) [11354 - 11416 (13.1 - 13.2%)] |
| ≥ 23 days | 7563 (10.1%) [7516 - 7610 (10.0 - 10.1%)] | 4718 (7.3%) [4625 - 4813 (7.1 - 7.4%)] | 7451 (8.6%) [7426 - 7474 (8.6 - 8.6%)] |
| ≥ 27 days | 5232 (7.0%) [5200 - 5264 (6.9 - 7.0%)] | 3301 (5.1%) [3225 - 3375 (5.0 - 5.2%)] | 4723 (5.5%) [4706 - 4740 (5.4 - 5.5%)] |
| Mean - Among Delayed | 8.06 [8.04 - 8.08] | 6.64 [6.60 - 6.68] | 8.16 [8.15 - 8.17] |
| Median - Among Delayed | 4 [4 - 4] | 2 [2 - 2] | 5 [5 - 5] |
| Mean - Everyone Included | 1.68 [1.68 - 1.69] | 1.20 [1.19 - 1.20] | 1.96 [1.96 - 1.97] |
| Median - Everyone Included | 0 [0 - 0] | 0 [0 - 0] | 0 [0 - 0] |

**Supplementary Table 8. Expanded simulation results for tuberculosis**

|  | **Algorithm 1** | **Algorithm 2 (alpha = 0)** | **Algorithm 2 (alpha = 1)** |
| --- | --- | --- | --- |
| **Number of Missed Opportunities (n 95% CI))** |  |  |  |
| Outpatient | 5750 (89.2%) [CI: 5724 - 5778 (88.8 - 89.7%)] | 5816 (90.3%) [CI: 5788 - 5846 (89.8 - 90.7%)] | 5708 (88.6%) [CI: 5684 - 5733 (88.2 - 89%)] |
| Inpatient | 324 (5.0%) [CI: 304 - 342 (4.7 - 5.3%)] | 315 (4.9%) [CI: 297 - 333 (4.6 - 5.2%)] | 327 (5.1%) [CI: 310 - 343 (4.8 - 5.3%)] |
| ED | 370 (5.7%) [CI: 348 - 390 (5.4 - 6.1%)] | 313 (4.9%) [CI: 291 - 334 (4.5 - 5.2%)] | 409 (6.3%) [CI: 391 - 425 (6.1 - 6.6%)] |
| Total | 6444 | 6444 | 6444 |
| **Number of Patients Experiencing Missed Opportunities (n (% of all patients) [95% CI])** |  |  |  |
| 0 | 454 (21.9%) [438 - 470 (21.1 - 22.7%)] | 748 (36.1%) [725 - 771 (35.0 - 37.2%)] | 367 (17.7%) [361 - 374 (17.4 - 18.0%)] |
| ≥ 1 | 1619 (78.1%) [1603 - 1635 (77.3 - 78.9%)] | 1325 (63.9%) [1302 - 1348 (62.8 - 65.0%)] | 1706 (82.3%) [1699 - 1712 (82.0 - 82.6%)] |
| ≥ 2 | 1309 (63.2%) [1290 - 1329 (62.2 - 64.1%)] | 1071 (51.7%) [1049 - 1092 (50.6 - 52.7%)] | 1391 (67.1%) [1375 - 1408 (66.3 - 67.9%)] |
| ≥ 3 | 1011 (48.8%) [992 - 1032 (47.9 - 49.8%)] | 861 (41.5%) [841 - 880 (40.6 - 42.5%)] | 1062 (51.2%) [1044 - 1081 (50.4 - 52.1%)] |
| ≥ 4 | 756 (36.5%) [736 - 775 (35.5 - 37.4%)] | 695 (33.5%) [675 - 714 (32.6 - 34.4%)] | 765 (36.9%) [747 - 784 (36.0 - 37.8%)] |
| ≥ 5 | 548 (26.4%) [529 - 566 (25.5 - 27.3%)] | 555 (26.8%) [537 - 574 (25.9 - 27.7%)] | 530 (25.6%) [513 - 547 (24.7 - 26.4%)] |
| Mean - Overall | 3.98 [3.94 - 4.02] | 4.87 [4.78 - 4.95] | 3.78 [3.76 - 3.79] |
| Median - Overall | 3 [3 - 3] | 4 [4 - 4] | 3 [3 - 3] |
| Mean - Outpatient | 3.55 [3.51 - 3.59] | 4.39 [4.31 - 4.47] | 3.35 [3.33 - 3.37] |
| Median - Outpatient | 3 [3 - 3] | 4 [3 - 3] | 3 [3 - 3] |
| Mean - Inpatient | 0.20 [0.19 - 0.21] | 0.24 [0.22 - 0.25] | 0.19 [0.18 - 0.20] |
| Median - Inpatient | 0 [0 - 0] | 0 [0 - 0] | 0 [0 - 0] |
| Mean - ED | 0.23 [0.22 - 0.24] | 0.24 [0.22 - 0.25] | 0.24 [0.23 - 0.25] |
| Median - ED | 0 [0 - 0] | 0 [0 - 0] | 0 [0 - 0] |
| **Durations of Delays (n (% of patients experiencing missed opportunities) [95% CI])** |  |  |  |
| ≥ 1 days | 1619 (100.0%) [1603 - 1635 (100.0 - 100.0%)] | 1325 (100.0%) [1302 - 1348 (100.0 - 100.0%)] | 1706 (100.0%) [1699 - 1712 (100.0 - 100.0%)] |
| ≥ 11 days | 1355 (83.7%) [1336 - 1374 (82.7 - 84.6%)] | 1024 (77.3%) [1001 - 1048 (75.6 - 79.0%)] | 1489 (87.3%) [1481 - 1498 (86.9 - 87.7%)] |
| ≥ 22 days | 1099 (67.9%) [1080 - 1120 (66.8 - 69.0%)] | 776 (58.6%) [754 - 801 (56.8 - 60.5%)] | 1267 (74.3%) [1257 - 1277 (73.8 - 74.8%)] |
| ≥ 33 days | 853 (52.7%) [833 - 871 (51.5 - 53.8%)] | 552 (41.7%) [529 - 575 (39.9 - 43.5%)] | 1026 (60.1%) [1015 - 1036 (59.6 - 60.7%)] |
| ≥ 45 days | 623 (38.5%) [603 - 643 (37.3 - 39.7%)] | 387 (29.2%) [368 - 407 (27.8 - 30.9%)] | 820 (48.1%) [809 - 831 (47.5 - 48.7%)] |
| ≥ 56 days | 459 (28.4%) [441 - 476 (27.3 - 29.3%)] | 261 (19.7%) [245 - 278 (18.4 - 21.0%)] | 579 (33.9%) [569 - 589 (33.4 - 34.5%)] |
| ≥ 67 days | 298 (18.4%) [285 - 312 (17.6 - 19.2%)] | 186 (14.1%) [172 - 201 (12.9 - 15.2%)] | 371 (21.7%) [363 - 379 (21.3 - 22.2%)] |
| ≥ 79 days | 173 (10.7%) [163 - 182 (10.1 - 11.2%)] | 122 (9.2%) [111 - 134 (8.4 - 10.2%)] | 197 (11.6%) [191 - 202 (11.2 - 11.9%)] |
| ≥ 90 days | 124 (7.7%) [117 - 131 (7.2 - 8.1%)] | 75 (5.7%) [67 - 84 (5.0 - 6.3%)] | 116 (6.8%) [112 - 119 (6.6 - 7.0%)] |
| Mean - Among Delayed | 39.86 [39.31 - 40.37] | 34.35 [33.52 - 35.15] | 44.47 [44.16 - 44.75] |
| Median - Among Delayed | 36 [34 - 36] | 28 [26 - 28] | 43 [42 - 43] |
| Mean - Everyone Included | 31.13 [30.64 - 31.59] | 21.95 [21.45 - 22.45] | 36.59 [36.30 - 36.85] |
| Median - Everyone Included | 25 [23 - 25] | 10 [9 - 11] | 33 [32 - 33] |

**Supplementary Table 9. Sensitivity analysis using all visits - simulation parameters and results of change-point analysis.**

|  | **Stroke** | **AMI** | **Tuberculosis** |
| --- | --- | --- | --- |
| **Change-point Model** | Prediction Bound | Prediction Bound | Linear Cubic |
| **Change-point** | 31 | 31 | 125 |
| **Delay Window** | [31, 1] | [31, 1] | [125, 1] |
| **Total Number of Missed Opportunities During Delay Window (% of all visits during delay window)** | 214,316 (22.7%) | 170,058 (22.9%) | 9,851 (39.4%) |

**Supplementary Table 10. Selected Simulation Results for sensitivity analysis using**

|  | **Stroke** | **AMI** | **Tuberculosis** |
| --- | --- | --- | --- |
| **Change Point (Start of diagnostic opportunity Window** | 31 | 31 | 125 |
| **Total Number of Missed Opportunities During Delay Window (% of SSD visits during delay window)** | 214,316 (22.75%) | 170,058 (22.94%) | 9,851 (39.41%) |
|  |  |  |  |
| **Percent of Missed opportunities in outpatient settings** |  |  |  |
| Algorithm 1 | 85.51 (85.40 - 85.62) | 85.86 (85.75 - 85.97) | 92.73 (92.37 - 93.08) |
| Algorithm 2 | 86.79 (86.66 - 86.91) | 86.79 (86.65 - 86.92) | 93.39 (93.02 - 93.78) |
| Algorithm 3 | 83.71 (83.61 - 83.80) | 84.43 (84.32 - 84.52) | 92.26 (91.95 - 92.60) |
| **Percent of missed opportunities in inpatient settings** |  |  |  |
| Algorithm 1 | 2.55 (2.50 - 2.61) | 2.61 (2.55 - 2.67) | 2.85 (2.62 - 3.09) |
| Algorithm 2 | 2.48 (2.43 - 2.53) | 2.57 (2.52 - 2.63) | 2.82 (2.62 - 3.01) |
| Algorithm 3 | 2.54 (2.49 - 2.59) | 2.65 (2.60 - 2.71) | 2.84 (2.64 - 3.06) |
| **Percent of Missed opportunities in ED settings** |  |  |  |
| Algorithm 1 | 11.94 (11.84 - 12.04) | 11.52 (11.43 - 11.63) | 4.42 (4.16 - 4.70) |
| Algorithm 2 | 10.73 (10.62 - 10.85) | 10.64 (10.52 - 10.76) | 3.79 (3.48 - 4.09) |
| Algorithm 3 | 13.75 (13.66 - 13.84) | 12.92 (12.84 - 13.01) | 4.89 (4.65 - 5.15) |
| **Percent of Patients Experiencing Missed Opportunities (95% CI)** |  |  |  |
| Algorithm 1 | 33.92 (33.84 - 33.99) | 28.88 (28.82 - 28.95) | 90.49 (89.77 - 91.17) |
| Algorithm 2 | 22.43 (22.34 - 22.51) | 20.36 (20.29 - 20.44) | 62.45 (61.07 - 63.97) |
| Algorithm 3 | 44.24 (44.20 - 44.29) | 36.48 (36.43 - 36.52) | 94.56 (94.36 - 94.79) |
| **Mean Number of Missed opportunities among patients missed (95% CI)** |  |  |  |
| Algorithm 1 | 1.72 (1.71 - 1.72) | 1.64 (1.63 - 1.64) | 5.25 (5.21 - 5.29) |
| Algorithm 2 | 2.60 (2.59 - 2.61) | 2.32 (2.31 - 2.33) | 7.61 (7.43 - 7.78) |
| Algorithm 3 | 1.32 (1.32 - 1.32) | 1.30 (1.29 - 1.30) | 5.03 (5.01 - 5.04) |
| **Mean duration (days) of missed opportunities among patients missed (95% CI)** |  |  |  |
| Algorithm 1 | 9.47 (9.45 - 9.49) | 8.44 (8.42 - 8.46) | 52.31 (51.60 - 53.00) |
| Algorithm 2 | 6.79 (6.75 - 6.83) | 5.85 (5.81 - 5.89) | 38.65 (37.35 - 39.90) |
| Algorithm 3 | 9.18 (9.17 - 9.18) | 8.22 (8.22 - 8.23) | 61.53 (61.18 - 61.88) |

**Supplementary Table 11. Sensitivity analysis using a non-linear (cubic) expected trend in SSD visits –** The total number of estimated missed opportunities are given using a linear baseline (used in primary analysis) versus a cubic baseline. The area under the curve (AUC), corresponding to the observed number of SSD visits during the diagnostic opportunity window, that represents missed opportunities is provided for both the linear and cubic trends; this is computed as the number of missed opportunities divided by the total number of SSD visits. For each disease, the use of a cubic baseline curve resulted in few er missed opportunities with missed opportunities capturing a smaller percentage of AUC during the diagnostic opportunity window.

| Condition | Change-point (based on linear trend prior | Missed Opportunities (using linear baseline) | Missed Opportunities (using cubic baseline) | Total SSD visits inside diagnostic opportunity window | | AUC % Using linear model | AUC % using cubic trend |
| --- | --- | --- | --- | --- | --- | --- | --- |
| TB | 114 | 6,444 | 5,928 | 11,082 | | 58.15 | 53.49 |
| AMI | 40 | 117,344 | 95,173 | 290,968 | | 40.33 | 32.71 |
| Stroke | 39 | 41,577 | 36,133 | 68,670 | | 60.55 | 52.62 |
|  |  |  |  | |  |  |  |
|  |  |  |  | |  |  |  |
|  |  |  |  | |  |  |  |
|  |  |  |  | |  |  |  |

**Supplementary Figure 1. Change-point detection approach results for stroke**


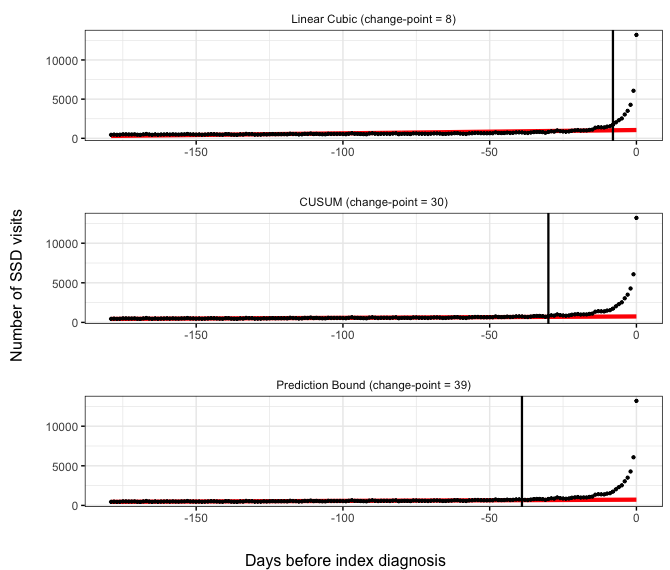


**Supplementary Figure 2: Change-point detection approach results for AMI**

**Supplementary Figure 3. Change-point detection approach results for tuberculosis**


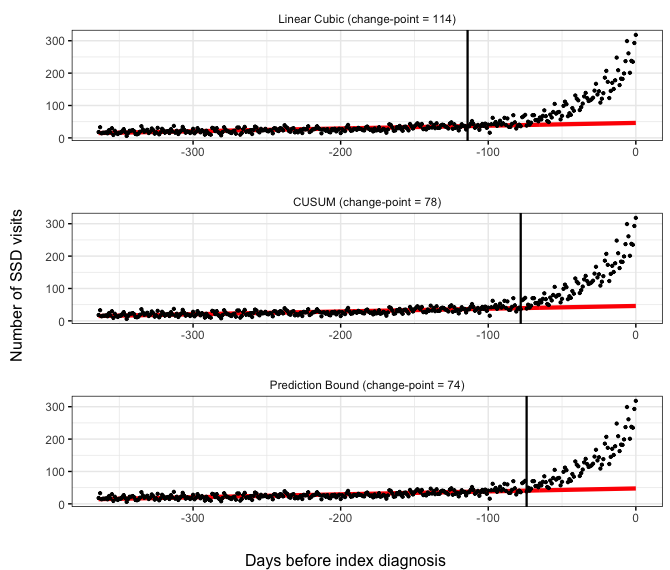

Supplement: Supplementary file 1 — Supplementary Material 1 [file 12911_2023_2148_MOESM1_ESM.docx]
